# Identification of Everyday Sounds Affects Their Pleasantness

**DOI:** 10.3389/fpsyg.2022.894034

**Published:** 2022-07-08

**Authors:** Laurie M. Heller, Jessica M. Smith

**Affiliations:** Auditory Lab, Department of Psychology, Carnegie Mellon University, Pittsburgh, PA, United States

**Keywords:** misophonia, sound category, sound emotion, causal properties, everyday sounds, sound identification, context effects, unpleasant sounds

## Abstract

This study examines the role of source identification in the emotional response to everyday sounds. Although it is widely acknowledged that sound identification modulates the unpleasantness of sounds, this assumption is based on sparse evidence on a select few sounds. We gathered more robust evidence by having listeners judge the causal properties of sounds, such as actions, materials, and causal agents. Participants also identified and rated the pleasantness of the sounds. We included sounds from a variety of emotional categories, such as Neutral, Misophonic, Unpleasant, and Pleasant. The Misophonic category consists of everyday sounds that are uniquely distressing to a subset of listeners who suffer from Misophonia. Sounds from different emotional categories were paired together based on similar causal properties. This enabled us to test the prediction that a sound’s pleasantness should increase or decrease if it is misheard as being in a more or less pleasant emotional category, respectively. Furthermore, we were able to induce more misidentifications by imposing spectral degradation in the form of envelope vocoding. Several instances of misidentification were obtained, all of which showed pleasantness changes that agreed with our predictions.

## Introduction

Everyday sounds play an important role in our lives by providing information about the events occurring in the world around us. For example, sounds help to keep us alive by warning us of approaching danger in our environment, especially in the absence of visual information such as in the dark or when we are asleep. Similarly, sounds signal the start of new events, causing us to divert our attention to sudden changes in sound (Neuhoff and Heller, 2015). Upon hearing a sound, we also cognitively infer features about its source and the physical event that produced it. Most sound-causing events are best described as a force applied to an object (or substance) causing it to vibrate. Since the sounds which humans hear are the result of the propagation of these vibrations (usually through air), sound provides vital information about the causal properties of the event. The properties considered in this study are: causal action (e.g., an impact), causal object (properties of the object that make the sound, such as a hollow drum and a stick), and causal agent (such as a person). There is evidence that people use causal properties when identifying sound sources. For example, people can identify causal actions (Guastavino, 2007; Lemaitre and Heller, 2012; Martín et al., 2015; Navolio et al., 2016; Lemaitre et al., 2018), causal materials and object properties (Arnott et al., 2008; Grassi et al., 2013; Lemaitre et al., 2018), causal sound source (Ballas, 1993), and causal agents (Caramazza and Shelton, 1998; Engel et al., 2009). While everyday sounds inform us about the environment, they are also qualitatively different than other common sounds, particularly social ones such as language and music. In contrast to language and music, everyday sounds are not as structured, tonal, and rhythmic. Instead, they contain more noise and randomness, which makes their acoustical features generally difficult to characterize (McDermott, 2012). Nonetheless, we have made progress in finding acoustic regularities in everyday sounds which can help discriminate their causal actions (Gygi et al., 2004; Heller and Sheikh, 2019).

While sounds inform us about events, it is also common for sounds to trigger emotional or physiological responses (Keil et al., 2007). Some sounds, such as a favorite piece of music, can evoke joy or pleasant chills (Blood and Zatorre, 2001; Barratt and Davis, 2015), while other sounds, such as crying, can evoke discomfort (Zald and Pardo, 2002; Anders et al., 2008; Grewe et al., 2011; Kumar et al., 2012; Ro et al., 2013). However, for a subset of people, certain common sounds elicit irritation, rage, or even panic (Edelstein et al., 2013; Rouw and Erfanian, 2018). Individuals who experience this type of debilitating response suffer from a sound intolerance disorder known as Misophonia (Jastreboff and Jastreboff, 2001; Swedo et al., 2022). Estimates of Misophonia prevalence range from about six to twenty percent of the population, depending on the criteria used, and Misophonia tends to impact more women than men (Potgieter et al., 2019). Misophonia has been characterized as a chronic disorder, and can be comorbid with other conditions, for example, obsessive–compulsive disorder, anxiety, and the personality trait of neuroticism (Cusack et al., 2018; Erfanian et al., 2019; Cassiello-Robbins et al., 2020, 2021; Çolak et al., 2021). Although Misophonia is similar to other sound intolerance disorders such as Hyperacusis, a number of researchers have made a strong case for Misophonia being a unique disorder in terms of its specific symptoms and neural responses (Edelstein et al., 2013; Cavanna and Seri, 2015; Kumar et al., 2017; Taylor, 2017; Brout et al., 2018). Although more than one set of criteria exists for Misophonia, including the Amsterdam Misophonia Scale (A-MISO-S), MisoQuest, Misophonia Questionnaire (MQ), and the Duke Misophonia Questionnaire, there is good agreement on the common trigger sounds (Schröder et al., 2013; Wu et al., 2014; Jager et al., 2020; Siepsiak et al., 2020; Naylor et al., 2021; Rosenthal et al., 2021). More specifically, common trigger sounds typically arise from everyday events which makes it particularly difficult to avoid triggers. Misophonia trigger sounds are often noises made by the body of another person, especially nasal and oral sounds, like slurping and chewing, and/or repetitive noises, such as keyboard typing or pencil tapping, but they are not confined to those categories (Enzler et al., 2021; Hansen et al., 2021). The person or context producing the sounds can affect the trigger’s potency. When in the presence of triggers, these sounds disturb mental faculties such as cognitive control and learning retention in misophonic individuals (Seaborne and Fiorella, 2018; Daniels et al., 2020). The prevalence of these triggers can cause people to avoid school, family, and friends. This avoidance can severely damage social interactions and overall quality of life.

Although misophonic triggers are well documented, there is no comprehensive or predictive explanation as to why certain sounds tend to become triggers. However, there is evidence in the literature that profound emotional responses to sound can be driven by the meanings and causes of the events that the sounds signify, rather than by the sounds’ acoustic qualities (Halpern et al., 1986; Brout et al., 2018; Edelstein et al., 2020). This claim is supported by the observation that the identification of a sound can change its perceived valence. Consider the example of scratching a slate blackboard. Listeners who are informed that the experiment used the sound of a scratched blackboard rated the sound as worse than participants who were not given this information (Ely, 1975; Halpern et al., 1986). It is worth asking whether this observation for a generally unpleasant sound generalizes over a wider range of sounds. There is one known example of a similar effect for a misophonic sound, in which human eating sounds are rated as more unpleasant if correctly categorized at the time of the rating (Edelstein et al., 2020). For all types of sounds, misophonic or not, it is useful to expand the repertoire of known instances in which misidentification of a sound changes its valence. Obtaining a larger number of examples will permit us to discover whether this effect is systematic and if so, how unpleasantness relates to the identification and causal properties of sounds.

Given that sound identification can influence the emotional response to a sound, it follows that the perception of causal properties should likewise affect the pleasantness of a sound. Yet, it is unknown how each of these causal properties contribute to a misophonic response. The types of sounds that have been found to precipitate misophonic responses are caused by a variety of actions (scraping, tapping, etc.), materials (metal, liquid, etc.), and agents (human, machine, etc.). For example, it may be the case that the same object produces either a disturbing or pleasant sound depending upon the action performed with it. One opportunity to study these questions is provided by misidentification of sounds because they reveal the effect of the meaning of a sound separate from its acoustics.

To create an experiment that produces misidentifications, we started with the observation that listeners naturally infer qualities about sounds when they hear them. Our study addresses this notion by asking listeners about the causal properties they hear in everyday sounds: actions, materials, and causal agents. In some instances, listeners might identify only some of the causal properties of a sound, but in others, they may infer multiple possible causes or misattribute a certain causal property. Considering that sound recognition is the endpoint of the auditory cognition process, causal properties such as actions and materials may be inferred (either correctly or incorrectly) regardless of whether the sound source is identified. Here we investigated whether misinterpretation of a sound’s cause altered its pleasantness. We hypothesized that a sound that is normally neutral should become more unpleasant if the causal action or material is heard incorrectly as being that of a more negative sound. In contrast, a sound that is normally unpleasant should become more positive if the cause of the sound is misheard as having a more neutral source.

We tested this hypothesis by including sounds that shared similar causal properties to encourage misidentification. The final set of everyday sounds belonged to one of the following categories: Neutral, Misophonic, Unpleasant, or Pleasant. Each Negatively valenced sound (from both Unpleasant and Misophonic categories) was paired with a Neutral sound; for example, the sound of *Slurping a beverage*, a Misophonic sound, was paired with the sound of a *Sink draining*, a Neutral sound. Listeners judged the pleasantness, causal actions, materials, and agents of all sounds before they attempted to identify any of them. Our population was not recruited or screened for any diagnosis, although we measured each listener’s self-reported tendency toward sound intolerance. In a second experiment, we address the fact that misidentification of causal properties of everyday sounds can happen due to a degradation of the acoustic signal. For example, distortion resulting from sensorineural hearing loss, hearing aids, and cochlear implants (CIs) may all degrade auditory inputs, thereby producing a higher rate of sound misidentifications. We tested whether such experimentally induced misidentifications would display the same effect on pleasantness seen in Experiment One and whether these acoustic distortions reduce pleasantness overall.

## Experiment 1

Our first Experiment examined the role of source identification in the pleasantness of everyday sounds. Naïve listeners assigned causal properties, such as materials, actions, and agents, to unidentified brief everyday sounds. We used prior research to sort these sounds into four emotional categories: Neutral, Pleasant, Unpleasant, or Misophonic (Cox, 2008; Enzler et al., 2021; pilot data). The possible causal actions were: crushing, scraping, tapping, ringing, blowing, puffing, suctioning, splashing, and flowing. The possible causal materials were: wood, metal, air, liquid, human body. The causal agents were living (either human or non-human) or non-living. Subsequently, listeners rated sound pleasantness, and lastly, they identified each sound from a closed set of possible labels. The stimulus set was constructed so that each Unpleasant and Misophonic sound had an action and material that was present in at least one Neutral sound. This design permitted the exploration of whether misattribution of a property was associated with a change in the pleasantness rating to match its attribution.

### Methods: Experiment 1

#### Participants

Recruitment for both experiments was conducted through Carnegie Mellon University’s (CMU) Psychology Department for course credit. Consent and procedures were approved by CMU’s Institutional Review Board. Participants under 18 years old or with abnormal hearing were excluded. All participants ranged in age from 18 to 22, with the majority being undergraduate students. Experiment 1 had 39 participants who passed the screening (21 male, 17 female, 1 other).

#### Stimuli

The stimuli were fourteen brief everyday sounds covering a range of categories, actions, and materials. The sounds were sorted into Neutral, Pleasant, Unpleasant, and Misophonic categories based on previous literature and preliminary tests (Cox, 2008; Enzler et al., 2021). Table 1 displays all fourteen sound stimuli, with their names, categories, and pair labels. See the next paragraph for a full discussion of the pair labels. Each sound was trimmed to have a duration between 1 and 5 s (see Supplementary Table S1). Sounds were diotic, 16-bit 44,100 Hz WAV files. Experiment 1 stimuli were matched based on perceptual loudness, which was equalized in two steps. First, the root mean square (RMS) of the sample amplitudes (−1 to 1) in each sound file was computed (RMS ranged from 0.00343 to 0.02386) and scaled to be equal. Second, listening in the lab determined that some sounds needed to be adjust downwards in level to match the loudness of the others. This process was done for each sound iteratively until they were agreed to match in loudness by a pilot test of three listeners. We obtained these sounds from various sources, including the Dataset of Environmental Sound Classification (ESC-50) (*Ringing church bells*, *Slurping beverage*, *Wind blowing*), in-house recordings (*Tool scraping*, *Squeezing spray bottle*, *Sink draining*, *Squeezing spray bottle*, *Chewing food*), and freesound.org (*Woodpecker tapping*, *Fork scraping plate*, *Ringing fire alarm*, *Nose sniffling*, *Clicking a pen*, *Stream flowing*) (Piczak, 2015).

**TABLE 1:** Fourteen sound stimuli utilized in Experiment 1 and Experiment 2.

Sound number	Sound name	Category	Pair label
1	Tool scraping	Neutral	N1
2	Ringing church bells	Neutral	N2
3	Squeezing spray bottle	Neutral	N3
4	Sink draining	Neutral	N4
5	Stirring cereal	Neutral	N5
6	Woodpecker tapping	Neutral	N6
7	Nose sniffling	Misophonic	M3
8	Slurping a beverage	Misophonic	M4
9	Chewing food	Misophonic	M5
10	Clicking a pen	Misophonic	M6
11	Fork scraping a plate	Unpleasant	U1
12	Ringing fire alarm	Unpleasant	U2
13	Wind blowing	Pleasant	P7
14	Stream flowing	Pleasant	P7

*The emotional category is noted for each sound, with six sounds belonging to the neutral category, four sounds belonging to the misophonic category, and two sounds each belonging to the unpleasant and pleasant categories. Each sound has a pair label to represent each pairing between a Neutral and Misophonic/Unpleasant (negative) sound that share at least one causal property. Each label is structured as C# (C, valence category; #, the pair number). In following tables, the pair label is added to each sound name.*

We paired the six Misophonic and Unpleasant sounds with each Neutral sound by shared causal properties. For each, the pair number and sound category is labeled by C# (C = sound category, either N for Neutral, M for Misophonic, U for Unpleasant, or P for Pleasant, and # = pair number). The intended pairings for Unpleasant sounds were the *Fork scraping plate* (U1) and *Tool scraping* (N1), which shared the scraping action and metal material, and the *Ringing fire alarm* (U2) and *Ringing church bells* (N2), which shared ringing action and metal material. These two intended pairs are denoted as Pairs 1 and 2, respectively. The pairings with Misophonic sounds were as follows: *Nose sniffling* (M3) and *Squeezing a spray bottle* (N3) shared puffing action and air material; *Slurping beverage* (M4) and *Sink draining* (N4) shared suctioning/flowing actions and liquid material; *Chewing food* (M5) and *Stirring cereal* (N5) shared crushing action; and *Clicking a pen* (M6) and *Woodpecker tapping* (N6) shared the tapping action. These pairings are denoted as Pairs 3–6. The two Pleasant sounds, *Stream flowing* and *Wind blowing* were not paired with a shared action or material and are both referred to as Pair 7 (P7). Supplementary Table S1 shows the sound pairings and their presumed overlapping causal properties.

#### Design

The five sections of experimental questions about sound events were: causal actions (nine items), causal materials (five items), causal agent (one item), pleasantness, and identification. These sections are displayed in Table 2, including specific details about each. These seventeen questions were divided into three blocks, with the fourteen sounds presented in random order within each block. The first block consisted of three matrices that contained the causal properties. Each sound was played once, with instructions that permitted replaying only if there was a technical difficulty in hearing the sound. On a single page, all matrices were presented beneath the sound clip and rated before moving on to the next sound. The action questions, presented in a matrix format, asked how likely it was that a particular action could have produced some, or all, of the sound. The action verbs were: crushing, scraping, tapping, ringing, blowing, puffing, suctioning, splashing, and flowing. This action set was based on previous studies (Lemaitre and Heller, 2012; Heller and Sheikh, 2019; Elizalde et al., 2021) and piloting; they were sound-generating action verbs that ensured every sound had at least one relevant action. We kept the number of verbs small by ensuring that they pertained to more than one sound and therefore were not equivalent to identifying a single sound source. The material matrix asked whether each material was directly involved in making the sound. The material words were: wood, metal, air, liquid, and human body. The causal agent question was a third matrix that asked how likely it was that the sound was caused by the actions of a living being, irrespective of it being a human or an animal. This animate agent question specifically asked whether a living source performing an action on an object caused the sound. This question allowed for the listener to indicate an animate cause (a human) directing the events (drumbeats) despite the core elements of the sound event being inanimate (a stick and a drum). In each of these rating questions, the instructions encouraged participants to give ratings greater than 1 for more than one question per sound. This instruction encouraged thoughtful responses and discouraged rating one action or material high and all others low.

**TABLE 2:** Main five survey sections of action, material, agent, pleasantness, and identification questions.

Survey section	Question(s)	Question type	Answer choice labels	Scale	# of Rating items	Rating items
Action	For each action listed below, how likely is it that the action is possibly producing some (or all) of the sound?	Matrix Rating	1 – definitely not producing the sound, 5 – definitely producing the sound	1–5	9	Crushing, Scraping, Tapping, Ringing, Blowing, Puffing, Suctioning, Splashing, Flowing
Material	For each material, how much does it describe the object directly making the sound?	Matrix Rating	1 – not present in sound at all, does not describe sound object, 5 – definitely present in the sound, does describe sound object	1–5,	5	Wood, Metal, Air, Liquid, Human Body
Agent	How likely is it that this sound was caused by the actions of a living being? (This includes actions performed by a person on an object.)	Single Rating	1 – non-living, 5 – living	1–5	1	Cause of action
Pleasantness	How pleasant is the sound to you?	Single Rating	−5 - very unpleasant, 0 - neutral, 5 - very pleasant	−5 –5	1	Pleasantness
Identification	For this sound, which noun and verb pair listed best identifies this sound?	Multiple-Choice	Chewing food, Clicking a pen, Fork scraping plate, Nose sniffling, Ringing church bell, Fire alarm ringing, Sink draining, Slurping beverage, Stream flowing, Stirring cereal, Squeezing spray bottle, Tool scraping, Woodpecker tapping, Wind blowing	–	–	–

*The first column displays each of the five survey question sections. The second column for each section displays the primary question asked during the section. The third column shows what type of questions were in the section. The fourth column details the specific answer choice labels provided on the questions to the participant, and the fifth column shows the general rating scale that participants had to choose from. The last two columns describe the number and identity of rating items that were ranked for each sound.*

The second experimental block contained questions about sound pleasantness. Sounds were presented one at a time on the page in random order. Pleasantness ratings for each sound were given via a slider scale ranging from –5 to 5, with endpoints labeled as very unpleasant, to very pleasant, with a 0 denoting neutrality.

In the third block, participants identified sounds in a closed-set task. Questions for each sound were presented one at a time, in random order. Each question only allowed for a single choice out of fourteen options, with each option correctly matching only one of the fourteen sounds. Each answer choice used both a noun and verb to describe the sound; this labeling method does not favor sounds that are described best by their action or by their object/source. The labels for the sounds were: chewing food, clicking a pen, fork scraping a plate, nose sniffling, ringing church-bell, fire-alarm ringing, sink draining, slurping beverage, stream flowing, stirring cereal, squeezing spray-bottle, tool scraping, wind blowing, and woodpecker tapping. This section came last so that these sound labels would not affect the judgments of causal properties or pleasantness.

To check for attentive online listening, a single oddball question was inserted between the first (causal action, material, and agent) and second (pleasantness) blocks of experimental trials. The oddball question contained a different sound and question compared to what was used in the rest of the experiment. After the last experimental block, participants were asked to recall the oddball question by answering a multiple-choice question about its contents.

For Experiment 1, the oddball question was a recording of a voice saying the word “rolling” and the question asked which verb was spoken during that trial. The answer choices included multiple options of verbs, including all nine causal action items except suctioning and flowing, and others (e.g., “rolling,” “clattering,” “calling,” “wailing,” “rotating,” “vibrating,” “dripping). The recall question at the end of the survey tasked the participant to choose the oddball sound from a list, including similar types of answers (e.g., “saying metal,” “saying wood,” “saying breaking”) or other environmental sounds (e.g., “dog barking,” “piano,” “people clapping,” “paper crumpling”).

#### Procedure

The survey typically took 30–40 min to complete via a secure online survey platform (Qualtrics). Participants were instructed to take the test in a private, quiet location of their choosing, to refrain from eating or chewing gum, and to wear headphones. Participants first completed a consent form and read the instructions for the experiment. They next completed questions about age, gender, hearing status, and sound tolerance. The sound tolerance items were taken directly from MisoQuest (Siepsiak et al., 2020), a questionnaire for Misophonia. They were asked to agree or disagree on a five-point Likert scale to the statements: “I find some sounds made by the human body unbearable,” and, “Some sounds bother me so much that I have difficulty controlling my emotions.” These two questions’ averaged score served as a measure of general Sound Intolerance. This subset of questions from this questionnaire was chosen because it assesses reactions to a broad range of sounds efficiently without containing wording that presumes the participant has sound tolerance issues. Given that our participants were from a general population, we avoided questionnaires that specifically assume sound issues (e.g., MQ, MAQ, A-MISO-S, “My sound issues currently make me feel helpless.”) (Rosenthal et al., 2021). A third question that was also related to Hyperacusis was also asked: “Most loud sounds cause me to experience unpleasant emotions.” Next, participants completed a volume calibration in which white noise was played; its volume started out at zero and then the listener slowly increased it to a comfortable level. Next, a headphone screening asked participants to correctly do a task on at least two out of three trials that required the use of headphones (Milne et al., 2021). Finally, there was a practice trial with the experimental questions using a sound that was not in the main experiment. After the practice trial, the experiment began.

#### Data Quality Criteria

Participant responses were removed from the analysis if: (1) they indicated that they did not have normal hearing; (2) they failed the headphone screening; or (3) they answered the oddball questions incorrectly. Due to survey programming, some participants were not asked to complete headphone screening trials; their data were included after verifying that there was no significant effect of the screening condition on causal property ratings. That is, the ratings of these participants were statistically indistinguishable from the screened participants (via an ANOVA that treated the headphone screening condition as a between-participants factor).

### Results: Experiment 1

#### Data Analysis

Analysis of each experiment proceeds in four steps: (1) causal properties of each sound, (2) pleasantness of the sound categories, (3) identification accuracy of the sound categories, and (4) how misidentification changes both causal properties and pleasantness. A subsequent section compares results between both experiments, including sound intolerance self-ratings. All ANOVAs are reported with Greenhouse–Geisser corrections regardless of whether sphericity assumptions are significantly violated, and all Intraclass Correlation Coefficients (ICC) are reported for Average Measures, random effects, and consistency.

#### Causal Properties

A repeated measures ANOVA treated Sounds as a factor with 14 levels and Causal Properties as a factor with 15 levels (nine actions, five materials, and 1 causal agent). Both factors produced significant main effects [Sound: *F*(8.2,303) = 11.8, *p* < 0.001, G-G Epsilon 0.631; *P*roperty: *F*(6.0,221.4) = 83.8, *p* < 0.001, G-G Epsilon 0.427] which significantly interact [Sound by Property: *F*(22.4,830) = 60.0, *p* < 0.001, G-G Epsilon 0.123]. There is no between-groups main effect of the dichotic headphone Screen [*F*(1,37) = 0.008, *p* = 0.93] nor is there an interaction. Therefore, all data were combined, and this between-participant factor was removed from further analyses. Table 3 presents the mean ratings for each property and sound. Median ratings show very similar patterns in Supplementary Tables S2, S3.

**TABLE 3A:** Mean causal action property ratings taken across all participants are indicated in each table entry, with rows corresponding to one of the fourteen sound tokens and columns corresponding to the nine causal actions.

Category	Sound name	Crushing	Scraping	Tapping	Ringing	Blowing	Puffing	Suctioning	Splashing	Flowing
Neutral	(N1) Tool scraping	1.2	4.8	1.2	1.3	1.3	1.1	1.1	1.1	1.1
Unpleasant	(U1) Fork scraping plate	1.2	5.0	2.1	1.3	1.1	1.0	1.1	1.0	1.1
Neutral	(N2) Ringing church bells	1.2	1.3	1.7	4.9	1.2	1.1	1.1	1.1	1.1
Unpleasant	(U2) Ringing fire alarm	1.0	1.1	1.8	5.0	1.1	1.0	1.1	1.0	1.0
Neutral	(N3) Squeezing spray bottle	1.6	2.5	1.3	1.0	1.9	2.0	2.0	1.6	1.4
Misophonic	(M3) Nose sniffling	1.2	1.4	1.0	1.0	3.4	3.7	2.4	1.1	1.1
Neutral	(N4) Sink draining	1.0	1.1	1.0	1.0	1.2	1.2	1.9	3.2	4.3
Misophonic	(M4) Slurping beverage	1.1	1.1	1.0	1.0	1.5	1.4	4.4	2.1	2.0
Neutral	(N5) Stirring cereal	1.8	1.5	2.4	1.1	1.1	1.1	1.7	3.0	2.2
Misophonic	(M5) Chewing food	2.7	3.9	1.3	1.1	1.2	1.2	1.2	1.1	1.1
Neutral	(N6) Woodpecker tapping	1.4	1.8	4.4	1.2	1.2	1.1	1.2	1.1	1.1
Misophonic	(M6) Clicking a pen	1.4	1.4	4.3	1.2	1.2	1.1	1.3	1.0	1.0
Pleasant	(P7) Wind blowing	1.1	1.3	1.1	1.1	4.5	2.5	1.4	1.1	2.1
Pleasant	(P7) Stream flowing	1.0	1.1	1.2	1.0	1.2	1.1	1.3	2.8	4.8

*The intended valence category (neutral, unpleasant, misophonic, or pleasant) is indicated in the far-left column. Properties judged to have the highest likelihood of being the cause of a sound are colored in blue (mean > 4.0). Entries colored in green indicate means >3 and <=4. Entries colored in yellow indicate means >2 and <=3.*

Overall, the causal property ratings were appropriate for each sound. Table 3 shows a heatmap of the average rating for each causal property and sound. Table 3A specifically shows the average action ratings per sound while Table 3B shows the average material and agent ratings per sound. The sounds that were paired with each other are in adjacent rows. This table shows that the task had face validity. Listeners agree on the causal properties; the average measure Intraclass Correlation Coefficient (ICC) was 0.987 [95% CI from 0.985 to 0.990, *F*(209,7942) = 79.5, *p* < 0.0001). Both the *Tool scraping* and the *Fork scraping plate* sounds were rated high on scraping and metal with an animate causal agent. Both the *Ringing church bells* and *Ringing fire alarm* sounds were rated high on ringing and metal. Both the *Squeezing spray bottle* and *Nose sniffling* were rated high for air, but only *Nose sniffling* was heard as caused by puffing and blowing from a body. Results suggested that the *Squeezing spray bottle* was heard as having some likelihood of being caused by scraping wood. The pair of *Sink draining* and *Slurping beverage* both had splashing and liquid properties but *Sink draining* had more flowing, while *Slurping beverage* had more suctioning. The pair of *Stirring cereal* and *Chewing food* differed, with the *Stirring cereal* being rated high on splashing and liquid whereas *Chewing food* was rated higher on scraping and wood. Both the *Woodpecker tapping* and *Clicking a pen* sounds were heard as tapping caused by an animate agent, but the materials were wood and metal, respectively. As expected, *Wind blowing* was rated high on blowing whereas *Stream flowing* was rated high on flowing. Because of the many *post hoc* comparisons implicit in a table of this size, significance levels are not indicated. Instead, an average standard error (SE) of 0.137 per table entry was computed by initially deriving the SE across all 39 participants for each property and sound combination before averaging across all 210 of those SEs (fifteen causal properties x fourteen sounds). For reference, the maximum SE of any single value in the table was 0.32. Note the N per entry is equal for all cells.

**TABLE 3B:** Mean causal material and causal agent property ratings taken across all participants are indicated in each table entry, with rows corresponding to one of the fourteen sound tokens and columns corresponding to the five materials and one agent.

Category	Sound name	Wood	Metal	Air	Liquid	Body	Agent
Neutral	(N1) Tool scraping	1.3	4.9	1.3	1.0	1.4	3.5
Unpleasant	(U1) Fork scraping plate	1.3	5.0	1.1	1.0	1.3	4.3
Neutral	(N2) Ringing church bells	1.3	5.0	1.8	1.1	1.2	3.0
Unpleasant	(U2) Ringing fire alarm	1.2	4.9	1.6	1.1	1.2	2.1
Neutral	(N3) Squeezing spray bottle	2.0	2.1	3.3	1.9	1.8	3.3
Misophonic	(M3) Nose sniffling	1.3	1.1	4.0	1.2	4.8	4.8
Neutral	(N4) Sink draining	1.1	1.2	1.3	4.9	2.3	3.5
Misophonic	(M4) Slurping beverage	1.0	1.1	1.7	4.5	4.3	4.7
Neutral	(N5) Stirring cereal	2.6	1.8	1.3	3.7	2.3	3.6
Misophonic	(M5) Chewing food	3.3	1.7	1.2	1.6	3.1	3.9
Neutral	(N6) Woodpecker tapping	4.2	1.9	1.3	1.1	1.7	3.8
Misophonic	(M6) Clicking a pen	2.1	3.5	1.3	1.2	2.1	4.0
Pleasant	(P7) Wind blowing	1.2	1.6	4.9	1.4	1.5	1.9
Pleasant	(P7) Stream flowing	1.1	1.2	1.3	5.0	1.9	2.1

*The intended valence category (neutral, unpleasant, misophonic, or pleasant) is indicated in the far-left column. Properties judged to have the highest likelihood of being the cause of a sound are colored in blue (mean > 4.0). Entries colored in green indicate means >3 and <=4. Entries colored in yellow indicate means >2 and <=3.*

#### Pleasantness

**FIGURE 1:**
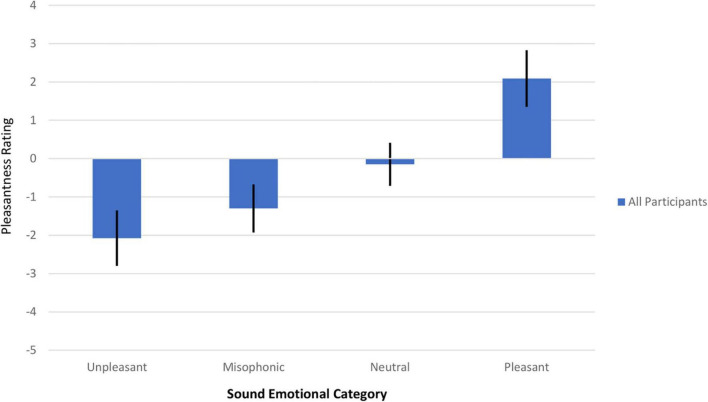
Mean pleasantness rating versus the sound emotional category for all participants in Experiment 1. A 95% confidence interval (*t*-test) is shown, represented by a thick black line. All four emotional categories are shown, in ascending order of average pleasantness (unpleasant, misophonic, neutral, and pleasant).

Although there was variation in the pleasantness of individual sounds within each emotional category, the mean valence of each category was ordered as expected, as shown in Figure 1. The far-left bar shows that the most unpleasant (and most negatively rated) category was the Unpleasant category (–2.08, 99%CI = 0.723), followed by the Misophonic category (–1.30, 99%CI = 0.627). The Neutral emotional category was rated close to the neutral zero rating (–0.150, 99%CI = 0.562) and the Pleasant category was rated positively (2.09, 99%CI = 0.739). The distribution of scores across participants did not violate normality or sphericity assumptions. A one-way ANOVA on the mean ratings showed that there was a main effect of Emotional Category on the pleasantness rating (*F*(2.6,100.1) = 69.5, *p* < 0.001, G-G Epsilon 0.88) with the caveat that the number of sounds in each category was unequal. Recall that the Unpleasant sounds were expected to be the most negatively rated given that our population did not overrepresent misophonic participants, but note that the Misophonic sounds were still negative, on average. Therefore, for some of our subsequent analyses on the effects of misidentification, we group the Unpleasant and Misophonic categories into a broader Negative valence group. Examining individual sounds instead of categories reveals that the most negatively rated single sound was *Nose sniffling* (mean of –2.56) and the most positively rated sound was a *Stream flowing* (mean of 3.02). Individual sounds remained in their *a priori* category regardless of their pleasantness rating. Most sounds were rated congruently with their *a priori* emotional category (their confidence interval of the average rating either was below zero, included zero, or above zero, if their emotional category was in the Negative, Neutral, or Positive valence group, respectively). The only exceptions were: two Neutral sounds (*Ringing church bells* rated positively and *Squeezing spray bottle* rated negatively) and two Misophonic sounds (*Clicking a pen* and *Chewing food* had negative averages but their CIs included zero). Nonetheless, our analysis kept sounds in their *a priori* categories.

#### Sound Identification Accuracy

Identification accuracy was computed by the percentage of participants who correctly selected the sound label out of the fourteen closed set options. Sound identification accuracy was high across all 39 participants (*M* = 90.1%, *SD* = 8.1%, *SE* = 1.5%, Median = 92.9%, Range = 61.5–100%). The overall identification accuracy for each sound, the sound it was most confused with, and how the valence of the emotional category shifted (upwards e.g., going from Negative to Neutral, or downwards, e.g., going from Negative to Neutral) is presented in Table 4; a complete confusion matrix is in Supplementary Table S4.

**TABLE 4:** Percentage of correct identification for each sound token and the sound (if applicable) it was most confused with across all participants in Experiment 1.

Sound name	Correct ID%	Most often confused with	Category shift
(N1) Tool scraping	61.54	Fork scraping plate (38.00%)	Neutral – Negative
(U1) Fork scraping plate	89.74	Tool scraping (8.00%)	Negative – Neutral
(N2) Ringing church bells	100.00	–	–
(U2) Ringing fire alarm	97.44	Ringing church bells (3.00%)	Negative – Neutral
(N3) Squeezing spray bottle	94.87	Tool scraping (5.00%)	–
(M3) Nose sniffling	100.00	–	–
(N4) Sink draining	89.74	Slurping beverage (5.00%)	Neutral – Negative
(M4) Slurping beverage	100.00	–	
(N5) Stirring cereal	64.10	Chewing food (13.00%)	Neutral – Negative
(M5) Chewing food	66.67	Tool scraping (23.00%)	Negative – Neutral
(N6) Woodpecker tapping	100.00	–	–
(M6) Clicking a pen	100.00	–	–
(P7) Wind blowing	100.00	–	–
(P7) Stream flowing	97.44	Sink draining (3.00%)	–

*Correct sound token names are in the first column while the most frequently perceived misidentification is in the second to last column. Based on the highest perceived misidentification, the last column denotes the objective shift in category and predicts how it affects pleasantness. The middle-left column contains the correct identification percentage for each sound. Each sound name also contains the sound’s pair label (emotional category and pair number).*

Our main hypothesis concerns sound tokens that were misidentified as a sound in another valence group. These emotional categories were defined *a priori* as indicated in Table 1, as well as the broader Negative valence group which was a combination of the Misophonic and Unpleasant categories. Because our hypothesis was specific to changes in valence, we did not analyze sounds that were misidentified as another sound in the valence group; for example, if a *Sink draining* was confused with *Stirring cereal*, this would be a confusion between two Neutral sounds and therefore would not be analyzed. However, if *Sink draining* was confused for *Slurping beverage*, which is a change across categories to a Negative valence sound (Misophonia emotional category), it would be a candidate for inclusion in the following analysis. A further criterion for inclusion was that this sound had to be misidentified across categories at least 10% of the time (i.e., by 4 of the 39 participants). There were four such sounds that met these criteria. These four qualifying sounds were subjected to subsequent analysis, as follows in the next section. There were two Neutral sounds misheard as something more Negatively valenced*: Tool scraping* and *Stirring cereal*. There were two Misophonic or Unpleasant sounds that were occasionally misheard as something more something more positively valenced (in both cases this was a neutral sound: *Chewing food*, and *Fork scraping plate*. These are “empirically discovered pairs” of misidentifications that can arise from the same sound; these are distinct from the planned pairs of sounds that had different sources and some overlapping causal properties. They can be noted in the confusion matrix in the Supplementary Table S4, which shows the actual sound in rows and the perceived sound category in columns. None of the sounds in the Pleasant category ended up qualifying for inclusion (because there were very few misidentifications involving those sounds). To clarify, in the following analysis, the perceived sound identity was determined from the identification data, but the emotional category was based on *a priori* predictions and the pleasantness data were not used to determine the emotional category.

#### Misidentifications

We used the opportunity provided by these empirically discovered misidentifications to test the prediction that pleasantness should be higher for the sounds heard in a more Positively valenced group than in a more Negatively valenced group, regardless of whether or not that identification is correct (see Table 5; a table of medians is provided in Supplementary Table S5). The mean pleasantness ratings for the four sounds identified in the more Positively valenced group were 1.35 points higher than the Negatively valenced group, which was a marginally significant difference [independent samples *t*(3) = 1.991, *p* < 0.07, one-tailed]. Because the number of misidentified sounds is small, the power is low (Cohen’s *d* = 1.4, Hedge’s correction = 1.6). In the following section, these data will be combined with more examples obtained from Experiment 2.

**TABLE 5:** Mean pleasantness ratings for the most frequently misidentified sounds in Experiment 1 as a function of how they are identified.

Sound name	Identification accuracy	Rating when perceived as unpleasant or misophonic	Identification accuracy	Rating when perceived as neutral or pleasant	Rating difference
Tool scraping	Incorrect	–0.2	Correct	–0.4	–0.2
Cereal stirring	Incorrect	–1.4	Correct	0.5	1.9
Chewing food	Correct	–0.9	Incorrect	–0.1	0.8
Fork scraping plate	Correct	–2.4	Incorrect	0.5	2.9
Average		−1.2		0.1	

**FIGURE 2:**
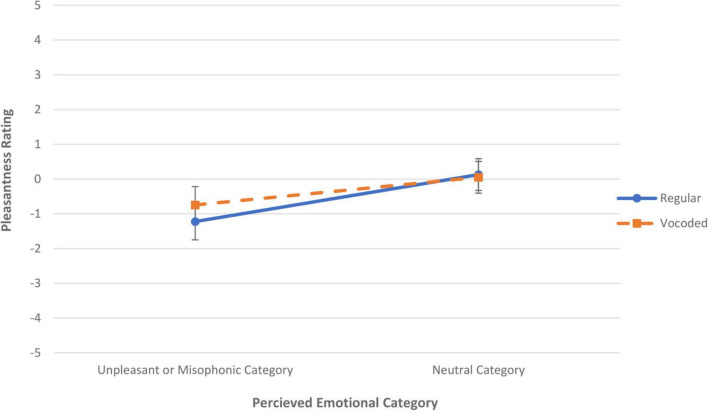
Mean pleasantness rating versus the perceived emotional category for both regular, non-vocoded sounds (solid blue line) and vocoded sounds (dashed orange line). Ratings of sounds misidentified within the same emotional category were subaveraged. The error bars denote standard error of the mean rating across sounds. If the sound was identified (regardless of correctness) as an item in one of our *a priori* negative categories (either a misophonic or unpleasant sound) then it contributed to a data point on the left, whereas if the sound was identified as an item in our neutral category (regardless of correctness), contributed to a data point on the right.

*For each sound stimulus, the sound token name is presented in the first column. The Identification accuracy column illustrates whether participants identified the sound correctly, i.e., with a label that fit into the same a priori emotional category (correct), or whether they misidentified the sound with a label that fit into a different a priori emotional category (incorrect). For the Correct entries, the mean pleasantness rating is taken across those participants who correctly identified the sound (less than 39 but always greater than 4, see Table 4); for the Incorrect entries, the mean pleasantness rating is taken across the 4 or more participants who made similar mistakes on the same sound. The bottom of the table shows the average mean pleasantness for when a sound is perceived as unpleasant or misophonic and when it is perceived as neutral or pleasant. The green and purple color code for these averages connects with the one seen in Supplementary Tables S4, S6. Here, a green box denotes when any category of sound is perceived as a sound in a neutral valence group. A purple box denotes when any category of sound is perceived as a sound in a negative valence group (either misophonic or unpleasant category). These average values can also be seen in the regular blue lines in Figure 2.*

## Experiment 2

Our second experiment addresses the fact that misidentification of causal properties of everyday sounds can happen due to a degradation of the acoustic signal. For example, distortion resulting from sensorineural hearing loss, hearing aids, and CIs may degrade auditory inputs, thereby producing a higher rate of sound misidentifications. We used psychoacoustically plausible signal degradation for two reasons: (1) it is a tool to produce more instances of misidentification to test our hypothesis, and (2) it is a step toward understanding how hearing loss can affect an individual’s positive or negative experience of everyday sounds. Therefore, we conducted a second experiment in which we spectrally degraded sounds processed by an envelope vocoder modulating a 16-channel noise. In prior experiments using envelope-modulated noise which removed frequency information but preserved temporal cues, the identifiability of roughly half of the sounds were impaired whereas half of the sounds still showed good identification (Gygi et al., 2004; Shafiro et al., 2011). We used the same procedure as Experiment 1 with vocoded versions of the sounds from Experiment 1.

### Methods: Experiment 2

#### Participants

There were 21 new young adult participants (10 male, 10 female, 1 other) in Experiment 2. Otherwise, the recruitment, consent process, and student population were the same as in Experiment 1.

#### Methods, Procedure, and Design

All methods and procedures were the same as for Experiment 1 except as noted here. The stimuli were 16-channel noise vocoded versions of the fourteen sounds used in Experiment 1. (AngelSim cochlear implant and hearing loss simulator, v1.08.01^1^; envelope detection cutoff frequency was 160 Hz with a 24 dB/oct slope; the signal was bandpass filtered into 16 logarithmically spaced channels that were bandpass filtered were analyzed; analysis and carrier filters used the Greenwood frequency function with low-pass and high-pass cutoffs of 200–7000 Hz with a 24 dB/oct slope.) Vocoding disrupts the spectral properties of the original sound but, by applying the amplitude envelope of the sound to noise, it preserves some of the temporal properties. The vocoded sounds were presented at the same RMS value as in Experiment 1. During the pre-trial instructions, participants listened to five examples of non-target sounds paired in their original and vocoded forms to familiarize themselves with the sound of vocoded sounds. All the participants were asked to complete the same headphone screening trials. In Experiment 2, the oddball trial contained audio asking participants to ‘Rate every action a 4’ while the visual format of the causal action response matrix looked the same as other trials. In the final recall question, participants indicated the spoken oddball sound they heard in a multiple-choice format (e.g., “rate every material a 2,” “skip this question,” etc.). Data from eleven out of 32 people were disqualified from Experiment 2 for failing any one of these criteria.

### Results: Experiment 2

#### Data Analysis

Analysis of Experiment 2 focuses primarily on the effects of identification on pleasantness. These data will also be integrated with several analyses that include data from both experiments.

#### Causal Properties

As with the regular sounds in Experiment 1, the causal properties of each sound were summarized as means. Listeners agreed on the causal properties [ICC = 0.936, 95%CI 0.922 to 0.948, *F*(209, 4180) = 15.6, *p* < 0.0001]. A repeated measures ANOVA on vocoded sounds treated Sounds as a factor with 14 levels and Causal Properties as a factor with 15 levels. Both factors produced significant main effects [Sound: *F*(6.9, 137) = 5.6, *p* < 0.001, G-G Epsilon = 0.529; Property: *F*(6.7,135) = 31.7, *p* < 0.001, G-G Epsilon = 0.481] that interact significantly [Sound by Property: *F*(16.3,325) = 13.4, *p* < 0.001, G-G Epsilon = 0.089].

#### Pleasantness

The average pleasantness of the vocoded sounds overall was –0.35. The average pleasantness for the six sounds in the Neutral category was –0.63 (95%CI = –1.18 to –0.076). The four sounds in the Misophonic category had an average pleasantness of –0.49 (95%CI = –1.10 to 0.120). The two sounds in the Unpleasant category had an average pleasantness average of –1.43 (95%CI = –2.20 to –0.66). The two sounds in the Pleasant category had the highest average pleasantness of 1.83 (95%CI = 0.93 to 2.74). Only the mean of the Pleasant category had confidence intervals that did not overlap with the other categories.

#### Sound Identification Accuracy

In Experiment 2, we found that sound identification of spectrally degraded sounds was modest for most sounds (*M* = 53.7%, *SD* = 33.4%). The average identification accuracy for the Neutral, Misophonic, Unpleasant and Pleasant categories, respectively, were 42.1% (95%CI = 35.5% to 48.6%), 83.3% (95%CI = 77.0% to 89.6%), 26.2% (95%CI = 16.4% to 36.0%), and 38.1% (95%CI = 29.6% to 46.6%). Only the mean of the Misophonic emotional category had confidence intervals that did not overlap with the other categories, showing a higher accuracy. Average identification accuracies for each vocoded sound, the sound it was most confused with, and how the valence of the emotional category shifted (e.g., going from Negative to Neutral or from Negative to Neutral) are presented in Table 6; a complete confusion matrix is in Supplementary Table S6.

**TABLE 6:** Percentage of correct identification for each sound token and the sound it was most confused with across all participants in Experiment 2.

Sound name	Correct ID%	Most often confused with	Category shift
(V. N1) Tool scraping	42.86	Fork scraping plate (24.00%)	Neutral – Negative
(V. U1) Fork scraping plate	42.86	Tool scraping (33.00%)	Negative – Neutral
(V. N2) Ringing church bells	38.10	Wind blowing (33.00%)	Negative – Neutral
(V. U2) Ringing fire alarm	9.52	Wind blowing (33.00%)	Negative – Neutral
(V. N3) Squeezing spray bottle	23.81	Tool scraping (33.00%)	–
(V. M3) Nose sniffling	95.24	Wind blowing (5.00%)	Negative – Neutral
(V. N4) Sink draining	28.57	Slurping beverage (29.00%)	Neutral – Negative
(V. M4) Slurping beverage	95.24	Sink draining (5.00%)	Negative – Neutral
(V. N5) Stirring cereal	23.81	Tool scraping, Squeezing spray bottle, Sink draining, Clicking a pen (14.00%)	Neutral – Negative
(V. M5) Chewing food	42.86	Tool scraping, Stirring cereal (14.00%)	Negative – Neutral
(V. N6) Woodpecker tapping	95.24	Tool scraping (5.00%)	–
(V. M6) Clicking a pen	100.00	–	–
(V. P7) Wind blowing	9.52	Stream flowing (81.00%)	–
(V. P7) Stream flowing	66.67	Sink draining (33.00%)	–

*Correct sound recordings names are in the first column while the most frequently perceived misidentification is in the second to last column. Based on the highest perceived misidentification, the last column denotes the objective shift in category and predicts how it affects pleasantness. The middle column contains the correct identification percentage for each sound. Each sound name includes a ‘V’ to signify that it is vocoded and its pair label (emotional category and pair number).*

#### Misidentifications

Pleasantness shifts mapped to misidentification are shown in Table 7; a table of medians is provided in Supplementary Table S7. There were seven sounds that were misidentified at least three times as a sound in a different valence group (10%, the same criterion number used in Experiment 1). The top four rows of the table show a Neutral category sound, such as a *Tool scraping* in row 1, incorrectly identified as an Unpleasant sound (such as *Fork scraping plate*); its mean rating when misidentified this way is shown in row 1, column 3. For comparison, the rating of that sound when it was correctly identified by other participants is shown in row 1, column 5. The bottom three rows of the table show the mean rating of a correctly identified Misophonic or Unpleasant sound (such as *Ringing fire alarm* in row 7, column 3). For comparison, the rating of that sound when it was incorrectly identified as a Neutral sound (such as *Sink draining* or *Wind blowing*) is shown in row 7, column 5. In all seven cases, the mean rating decreases between column 3 and 5. In all cases, pleasantness increased or decreased as predicted when a sound group changed from Negative to Neutral, or from Neutral to Negative, respectively. The mean pleasantness ratings for the seven sounds identified in the more Positively valenced group were 0.95 points higher than the Negatively valenced group, which was a significant difference [*t*(6) = 4.102, *p* < 0.003, one-tailed, Cohen’s *d* = 0.61, Hedge’s correction = 0.66].

**TABLE 7:** Mean pleasantness ratings for the most frequently misidentified sounds in Experiment 2 as a function of how they are identified.

Sound name	Identification accuracy	Rating when perceived as unpleasant or misophonic	Identification accuracy	Rating when perceived as neutral or pleasant	Rating difference
(V) Tool Scrape	Incorrect	–0.8	Correct	–0.4	0.4
(V) Squeezing spray bottle	Incorrect	–1.0	Correct	0.1	1.1
(V) Sink draining	Incorrect	–1.5	Correct	0.5	2.0
(V) Stirring cereal	Incorrect	–1.0	Correct	0.4	1.4
(V) Chewing food	Correct	0.8	Incorrect	1.5	0.7
(V) Fork scraping plate	Correct	–2.0	Incorrect	–1.3	0.7
(V) Ringing fire alarm	Correct	–1.5	Incorrect	–1.2	0.3
Average		−1.0		−0.1	

*For each sound stimulus, the sound token name is presented in the first column, with an added ‘V’ to signify the sound is vocoded. The Identification accuracy column illustrates whether participants identified the sound correctly, i.e., with a label that fit into the same a priori emotional category (correct), or whether they misidentified the sound with a label that fit into a different a priori emotional category (incorrect). For the correct entries, the mean pleasantness rating is taken across those participants who correctly identified the sound (less than 21 but always greater than 3, see Table 6); for the incorrect entries, the mean pleasantness rating is taken across the 3 or more participants who made similar mistakes on the same sound. The green and purple color code for these averages connects with the one seen in Supplementary Tables S4, S6. Here, a green box denotes when any category of sound is perceived as a sound in a Neutral valence group. A purple box denotes when any category of sound is perceived as a sound in a Negative valence group (either misophonic or unpleasant category).*

Because all four misidentified sounds in Experiment 1 also appeared in Experiment 2, it was possible to combine sounds across the two studies for comparison and analysis. Figure 2 plots the mean valences for these four sounds as a function of their perceived sound category valence; the blue solid line indicates results from the regular (non-vocoded) stimuli in Experiment 1 and the orange dashed line indicates results from the vocoded stimuli in Experiment 2. This graph shows that the valence increases as the misidentification changes the category for both experiments. An ANOVA with factors of Vocoding and Valence of Perceived Category shows a main effect of Perceived Category [*F*(1,6) = 9.12, *p* < 0.02], but there is no main effect of Vocoding and no significant interaction between Vocoding and Perceived Category.

### Comparison Across Experiments 1 and 2 for All Sounds

#### Sound Intolerance Self-Rating

The distributions of Sound Intolerance scores are shown in the blue bars for Experiment 1 and in the orange bars for Experiment 2 in Supplementary Figure S1. Only five of our 60 participants earned a score of 4.5 or greater. In this section comparing experiments, Sound Intolerance scores are included as a covariate in the omnibus analysis to assess whether the variation within this unscreened population could account for variation in the property and pleasantness ratings.

#### Causal Properties

In an ANCOVA that included Vocoding as a between-group factor, Sound Intolerance rating as a covariate, and Sound token and Causal Properties as within-group factors, Vocoding (regular vs. vocoded stimuli) had a significant main effect on average Property ratings [*F*(1,57) = 4.6, *p* < 0.04] as well as a two-way interaction with the within-group factors of Sound [*F*(9.5,541) = 2.59, *p* < 0.002], Causal Property [*F*(7.6,433) = 12.6, *p* < 0.001], and a three-way interaction with the same factors [*F*(182,10374) = 10.5, *p* < 0.001]. Sound Intolerance rating had no main effect or interaction. There was a main effect of sound [*F*(9.5,541) = 3.0, *p* < 0.001, G-G Epsilon = 0.730], Property [*F*(7.6,433) = 12.6, *p* < 0.001, G-G Epsilon = 0.543], and a Sound by Property interaction [*F*(32,1841) = 7.06, *p* < 0.001].

The sound pairs had causal property ratings that were mostly consistent with the regular, non-vocoded sounds [ICC = 0.808, 95%CI 0.749–0.854, *F*(209,4180) = 5.2, *p* < 0.0001], with the average changes described below. After vocoding, both the *Tool scraping* and the *Fork scraping plate* sound decreased on scraping (decreased by 0.8 and 1.5, respectively) and metal (decreased by 2.7 and 2.4, respectively). Only *Fork scraping plate* decreased in animate causal agent (decreased by 1). *Ringing church bells* and *Ringing fire alarm* sounds were rated less robustly in ringing (decreased by 3.3 and 3.5, respectively) and metal (by 2 and 3.1, respectively). While the *Nose sniffling* maintained a high rating for air, puffing, and body, *Squeezing spray bottle* mildly decreased in all three of these properties (by 0.8, 0.6, and 0.4) while increasing its tendency to be heard as scraping wood (by 0.9). Both *Sink draining* and *Slurping beverage* maintained their ratings on splashing and liquid. *Stirring cereal* was still rated high on splashing and liquid whereas *Chewing food* was rated lower on scraping (decreased by 0.9) and body (by 1.4). Both the *Woodpecker tapping* and *Clicking a pen* sounds maintained a similar pattern to the regular sounds, with highest ratings on tapping and animate agent (within 1 point difference). While *Stream flowing* maintained a high rating on flowing and liquid, *Wind blowing* had a large increase in rating for both properties (increased by 2.6 and 3.6, respectively) and a large decrease in ratings for blowing and air ratings (both decreased by 3.1).

#### Pleasantness of Spectrally Degraded Sounds

On average, vocoded sounds were rated more neutrally; the Negatively valenced sound categories were rated as less unpleasant than in Experiment 1, whereas the Pleasant sound category was rated as less pleasant than in Experiment 1. Accordingly, an ANOVA showed no significant main effect of Vocoding, but there was a significant main effect of the Emotional Category [*F*(2.4,140.3) = 71.7, p < 0.001, G-G Epsilon = 0.806] and there was an interaction between Vocoding and Emotional Category [*F*(2.4,140.3) = 2.94, *p* < 0.05].

**FIGURE 3:**
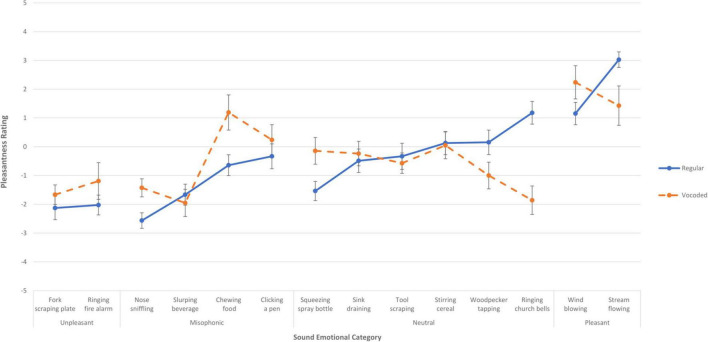
Mean pleasantness rating for each of the fourteen sounds when presented in Experiment 1, with no vocoding (solid blue lines), and when presented in Experiment 2 as vocoded (dashed orange lines). The error bars indicate the standard error of the mean across the sounds. Each of the fourteen sounds is plotted in its *a priori* emotional category, with the far-left, left, right, and far-right denoting the categories of unpleasant, misophonic, neutral, and pleasant. The more pleasant a sound is rated, the higher on the y axis it is placed.

To assess the effect of vocoding on pleasantness for individual sounds (rather than sound categories), an ANCOVA was done using the factors of Vocoding and Sound token and a covariate of Sound Intolerance self-rating. There was a main effect of Sound on pleasantness [*F*(9.5,542) = 2.57, *p* < 0.006] (G-G Epsilon = 0.723). Vocoding interacted with Sound [*F*(9.5,542) = 4.93, *p* < 0.001]. There was no main effect of Sound Intolerance nor did it interact. Relative to Experiment 1, pleasantness ratings were mostly unchanged; although a few sounds did not have overlapping standard error bars, this was equally distributed for both positive and negative shifts in pleasantness. Figure 3 shows the mean pleasantness ratings for each individual sound when it was presented as a regular sound in Experiment 1 (solid blue lines) and when it was spectrally degraded via vocoding in Experiment 2 (dashed orange lines); a similar figure showing medians is provided in Supplementary Figure S2. The blue lines grouping the two regular Unpleasant sounds in the far-left region of the figure show that these sounds are rated more unpleasant, on average, than the regular Neutral and Misophonic sounds, with the orange lines showing that the vocoded versions were slightly less negative on average (but all error bars overlap). The blue lines grouping the four regular Misophonic sounds in the left-middle region of the figure show that these sounds overlap in range with the Unpleasant sounds and the lower end of the regular Neutral sounds. The orange lines show that the vocoded Misophonic sounds are not consistently lower on average than the regular Misophonic sounds: Vocoded pleasantness increased beyond the error bars of *Chewing food* and *Nose sniffling*. The blue lines grouping the six regular Neutral sounds in the right-middle region of the figure show pleasantness ratings varying around the neutral point, ranging from -1.53 to 1.18, with the orange lines showing that the vocoded versions had no systematic effect on pleasantness. While the *Ringing church bells* and *Woodpecker tapping* sounds were less pleasant on average when vocoded, the *Squeezing spray bottle* was more pleasant when vocoded. The blue lines grouping the two regular Pleasant sounds on the far-right of the figure show that the Pleasant sound category was rated more highly than the other sounds on average. Vocoding reversed the pleasantness for the two sounds, by increasing beyond the regular condition’s error bars for *Wind blowing* while decreasing below the regular condition’s error bars for *Stream flowing*.

#### Identification

**FIGURE 4:**
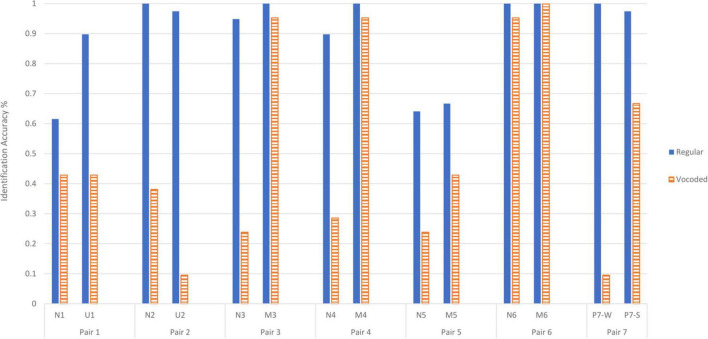
Mean identification accuracy for each of the fourteen sounds in their pairs for regular sounds in Experiment 1 (solid blue bars) and vocoded sounds in Experiment 2 (striped, orange bars). Each sound name is replaced with its pair label, with P7-W denoting *Wind flowing* and P7-S denoting *Stream flowing.* The higher the identification accuracy for a particular sound, the higher on the y axis it is placed.

The reduction in change in identification accuracy between Experiment 1 and 2 is evident in Figure 4. It displays percent accuracy as a function of each of the individual sound pairs for both Experiment 1 and 2. The process of vocoding produced an effect on identification in an ANOVA that treated the individual sounds as a within-participants variable and the two studies as a between-participants variable (regular or vocoded stimuli). Accuracy of 90.1% for regular sounds was higher than the accuracy of 53.6% for vocoded stimuli [*F*(1,58) = 228.4, *p* < 0.001]. There was also a main effect of Sound token [*F*(6.73,390.4) = 22.7, *p* < 0.001, G-G epsilon = 0.518] and an interaction between Vocoding and Sound [*F*(6.73,390.4) = 13.8, *p* < 0.001]. An ANOVA that had a within-subjects factor of Emotional Category and a between-subjects factor of Vocoding show that accuracy was significantly different for the different Emotional Categories [*F*(2.68,155.6) = 27.6, *p* < 0.001, G-G epsilon = 0.894]. Emotional Category interacts with Vocoding [*F*(2.68,155.6) = 31.9, *p* < 0.001]. An examination of the mean changes in accuracy between regular and vocoded sounds shows that the Neutral category significantly decreases from 85.0% (95%CI = 80.2% to 89.8%) to 42.1% (95%CI = 35.5% to 48.6%). For the Misophonic category, the accuracy does not reliably decrease from 91.7% (95%CI = 87.0% to 96.3%) to 83.3% (95%CI = 77.0% to 89.6%). The Unpleasant category accuracy significantly decreased from 93.6% (95%CI = 86.4% to 100.8%) to 26.2% (95%CI = 16.4% to 36.0%). The Pleasant category accuracy significantly decreased from 98.7% (95%CI = 92.5% to 105.0%) to 38.1% (95%CI = 29.6% to 46.6%). Despite these vocoding-induced changes in identification accuracy within categories, they did not correspond systematically to similar changes in pleasantness ratings.

## Discussion

Sound identification can influence sound pleasantness in ways that generalize across sounds. We were able to predict which direction pleasantness ratings should change based on which misidentifications were made. In order to produce misidentifications, we utilized sounds with similar causal properties in Experiment One, and we utilized spectral degradation in Experiment Two. Listeners rated the causal properties of these sounds so that we could assess whether the misidentifications were in fact based on these properties. We found that causal properties were reliably conveyed.

Sound identification rates for Experiment 1 were reasonably high at 90%. This outcome was expected because small closed-set tasks produce better performance than tasks with more options. The purpose of keeping this task relatively easy in Experiment 1 was to compare to performance in Experiment 2 on spectrally degraded sounds. We expected the spectrally degraded stimuli in Experiment 2 to impair identification. It did, but average identification was still at 53%.

The spectral degradation introduced by vocoding does not inherently make sound more unpleasant. In fact, it seems to make sounds more neutral, for both Positive and Negative valence groups. This result may be a consequence of the high rate of misidentification and uncertainty about the sounds’ causes. The misidentifications caused by vocoding helps to elucidate the relationship between causal properties and unpleasantness and can provide a baseline for future studies on the effects of hearing impairment.

Our goal in causing misidentifications was to use a principled approach to provide additional evidence for the importance of source identification on a sound’s unpleasantness. Our result is consistent with Edelstein et al. (2020) who showed an effect of identification on the emotional response to the sound of eating an apple. We note that our methodology differed from Edelstein et al. (2020) in a few ways. Our participants completed a single rating of the pleasantness of our sounds before they began identifying any sounds (with closed-set labels). In contrast, the participants in Edelstein et al. (2020) identified each sound’s category during two of the three trials in which they rated the sound’s valence. It is possible that different trial structures could alter the response to the valence task. For instance, the temporal proximity of the identification and valence tasks could increase the salience of a sound’s identity relative to when valence is the sole focus. It is also possible that rating the same sound three times in a row can have an effect. Another difference is that our participants heard a greater variety of sounds, about half of which were potentially unpleasant or misophonic. Finally, we conducted a normative population study rather than recruiting people with sound intolerance. Despite the methodological differences, our two studies reached similar conclusions about the importance of the relationship between sound identification and the emotional response to sound.

Because this was a normative study of a student population, comparisons with studies targeting misophonic populations are ventured with caution. We did seek to connect this study with others by asking whether the observed variation in self-rated intolerance levels could account for variance in sound pleasantness within our population, but no relationship was found in our data. It is possible that a stronger relationship would be seen with more severe intolerance; however, there were not enough participants to reliably analyze such a subpopulation. It is also possible that a more complete self-report of sound intolerance would reveal something not found here. The remaining questions about how the sound properties and pleasantness differ between populations could be addressed in a future study that targets more participants with sound intolerance issues such as Misophonia.

Within our population there are informative patterns of variation in sound properties and emotional categories across sounds. As a caveat, this study was not designed to test a correlation between causal properties and emotions. If such a correlation does exist in the natural world, we disrupted this regularity by setting up the paired sounds that had similar actions and materials but different emotional categories. By this same logic, if we do find a relationship between certain causal properties and their category, it cannot be viewed as causal. Surely, this study’s stimuli overrepresented certain properties in our selection process. Nonetheless, it should be informative to mention some perceptual patterns that did emerge. The Pleasant sounds, as a group, were rated relatively higher on blowing and flowing (i.e., compared to the other three categories). Conversely, Pleasant sounds were rated relatively lower on “animate causal agent.” Ratings tended to be high on metal and scraping for unpleasant sounds. Finally, as expected, ratings for a human body as a material were especially high for the Misophonic category.

Obtaining a likelihood rating for a range of causal properties for every sound is time-consuming in an experiment. This approach limited the number of questions and sounds that could be heard by the same listener within a short time span. But causal information is helpful when searching for a way to generate, predict, or even resolve sound confusions (e.g., Elizalde et al., 2021). For example, there was a hint in the data that the recording of *Squeezing spray bottle* was sometimes heard as being caused by scraping wood. This suggest that multiple actions can be interpreted from the same acoustic stimulus. A better understanding of how causal properties of events are perceived through sound might lead to insights into how and why sounds produce emotions. If scientists can decode the clues given by the subset of sounds that are common misophonic triggers, this may shed light on the root cause of why certain people develop Misophonia and could help lead to more effective treatments.

Our study looked at the impact of identification on the emotional response to sounds, but it is also true that this emotional response to a sound is related to its perceptual judgments and sound discrimination (Bergman et al., 2016). Vitale et al. (2020) noted that more than one type of measure is necessary to characterize the emotional response to sounds, and misophonic responses can also be triggered by non-auditory stimuli. By addressing these issues and beyond, future research may extend to applications beyond Misophonia, such as finding ways to make auditory environments more pleasant for everyone (DeLoach et al., 2015).

## Data Availability Statement

The raw data supporting the conclusions of this article will be made available by the authors, without undue reservation.

## Ethics Statement

The studies involving human participants were reviewed and approved by Carnegie Mellon University Institutional Review Board. Written informed consent for participation was not required for this study in accordance with the national legislation and the institutional requirements.

## Author Contributions

Both authors listed have made a substantial, direct, and equal intellectual contribution to the work, and approved it for publication.

## Conflict of Interest

The authors declare that the research was conducted in the absence of any commercial or financial relationships that could be construed as a potential conflict of interest.

## Publisher’s Note

All claims expressed in this article are solely those of the authors and do not necessarily represent those of their affiliated organizations, or those of the publisher, the editors and the reviewers. Any product that may be evaluated in this article, or claim that may be made by its manufacturer, is not guaranteed or endorsed by the publisher.

## Footnotes

1
http://www.tigerspeech.com/angelsim/angelsim_about.html
