# Supplementary material for: Identification of Everyday Sounds Affects Their Pleasantness
Source: Front Psychol. 2022 Jul 8;13:894034. doi: 10.3389/fpsyg.2022.894034 (PMC9347306; doi:10.3389/fpsyg.2022.894034)
Supplement: Supplementary file 1 [file Data_Sheet_1.ZIP › Supplemental Material/FigureS1.pdf]

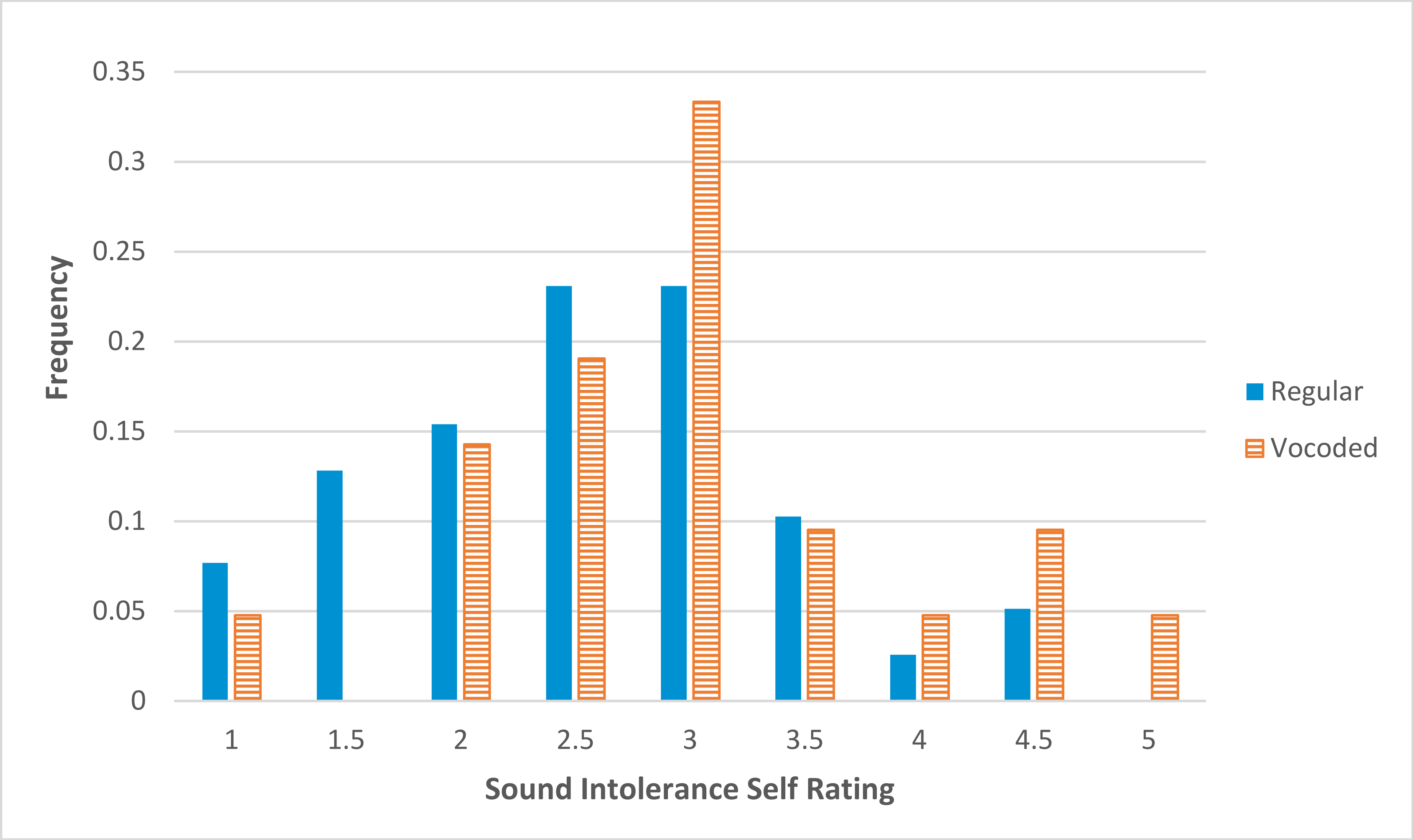

Figure S1: Histogram of average Sound Intolerance scores for all participants in Experiment 1 and 2. The solid blue bars denote the data from Experiment 1 using regular, non-vocoded sounds and the striped-orange bars denote the data from Experiment 2 using vocoded sounds. These scores were created by averaging the ratings from two questions about misophonic tendencies. The mean and median dislike score for Experiment 1 was 2.54 and 2.50, respectively (SD 0.88) with a distribution that did not significantly deviate from normality (Kolmogorov-Smirnov normality test D(39)=0.124, p>0.1).
