# Supplementary material for: Identification of Everyday Sounds Affects Their Pleasantness
Source: Front Psychol. 2022 Jul 8;13:894034. doi: 10.3389/fpsyg.2022.894034 (PMC9347306; doi:10.3389/fpsyg.2022.894034)
Supplement: Supplementary file 1 [file Data_Sheet_1.ZIP › Supplemental Material/FigureS2.pdf]

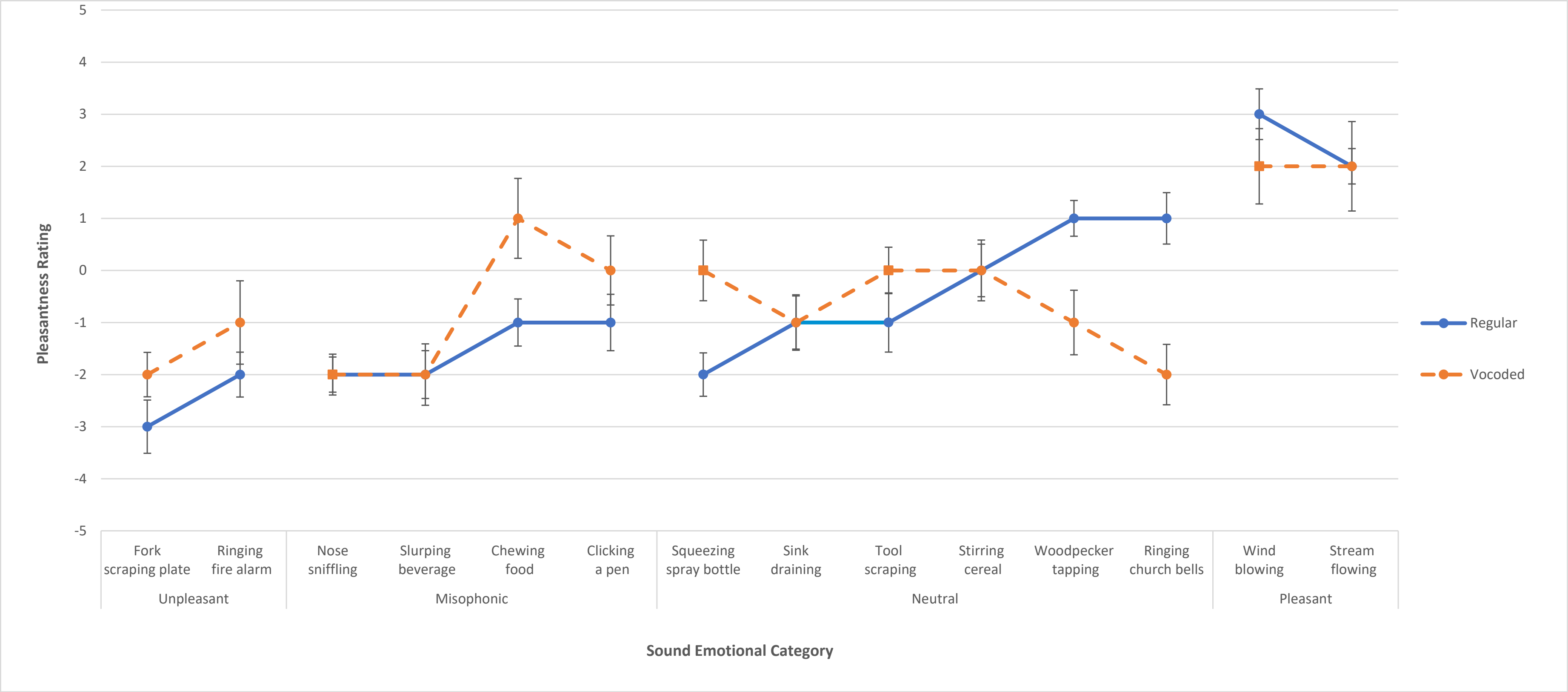

Figure S2: Median pleasantness rating for each of the fourteen sounds when presented in Experiment 1, with no vocoding (solid blue lines), and when presented in Experiment 2 as vocoded (dashed orange lines). The error bars indicate the standard error of the mean across the sounds. Each of the fourteen sounds is grouped into an emotional category, with the far-left, left, right, and far-right denoting the categories of Unpleasant, Misophonic, Neutral, and Pleasant. The more pleasant a sound is rated, the higher on the y axis it is placed.
