# Supplementary material for: Identification of Everyday Sounds Affects Their Pleasantness
Source: Front Psychol. 2022 Jul 8;13:894034. doi: 10.3389/fpsyg.2022.894034 (PMC9347306; doi:10.3389/fpsyg.2022.894034)
Supplement: Supplementary file 1 [file Data_Sheet_1.ZIP › Supplemental Material/TableS1.pdf]

Pair Number	Sound name	Sound Duration (s)	Predicted Shared Properties
**Pair 1**	N1. Tool scrape	1.36	Scraping, Metal, living Agent
	U1. Fork scraping plate	3.85	
**Pair 2**	N2. Ringing church bells	3.57	Ringing, Metal
	U2. Ringing fire alarm	2.62	
**Pair 3**	N3. Squeezing spray bottle	2.40	Puffing, Air, living Agent
	M3. Nose sniffling	3.44	
**Pair 4**	N4. Sink draining	2.73	Suctioning, Liquid, living Agent
	M4. Slurping a beverage	2.74	
**Pair 5**	N5. Stirring cereal	3.67	Crushing, living Agent
	M5. Chewing food	3.96	
**Pair 6**	N6. Woodpecker tapping	2.24	Tapping, living Agent
	M6. Clicking a pen	4.77	
**Pair 7**	P7. Wind blowing	5.00	-
	P7. Stream flowing	4.81	

Table S1: Duration and property information for each sound pair. The first two columns, Pair Number and Sound name, show which sounds are in each pair, as well as their pair label. This label, structured as C# (C= emotional category, # = pair number) also gives the emotional category of each sound, either Neutral (N), Unpleasant (U), Misophonic (M), or Pleasant (P). The column Sound Duration gives the length of each sound stimulus, in seconds. The Average Pair Duration gives the average of the duration for each sound pair, and Difference in Duration shows the absolute value of duration difference between the two paired sounds. The last column, Predicted Shared Properties, displays the properties the sounds were predicted to share, and thus were paired together. The final ratings found in Experiment 1 did not follow all the predicted properties listed here.
