# Supplementary material for: Identification of Everyday Sounds Affects Their Pleasantness
Source: Front Psychol. 2022 Jul 8;13:894034. doi: 10.3389/fpsyg.2022.894034 (PMC9347306; doi:10.3389/fpsyg.2022.894034)
Supplement: Supplementary file 1 [file Data_Sheet_1.ZIP › Supplemental Material/TableS3.pdf]

Category	Sound Name	Wood	Metal	Air	Liquid	Body	Agent
Neutral	N1. Tool scraping	1.0	5.0	1.0	1.0	1.0	4.0
Unpleasant	U1. Fork scraping plate	1.0	5.0	1.0	1.0	1.0	5.0
Neutral	N2. Ringing church bells	1.0	5.0	1.0	1.0	1.0	3.0
Unpleasant	U2. Ringing fire alarm	1.0	5.0	1.0	1.0	1.0	1.0
Neutral	N3. Squeezing spray bottle	2.0	1.0	4.0	1.0	1.0	3.0
Misophonic	M3. Nose sniffling	1.0	1.0	4.0	1.0	5.0	5.0
Neutral	N4. Sink draining	1.0	1.0	1.0	5.0	2.0	3.0
Misophonic	M4. Slurping beverage	1.0	1.0	1.0	5.0	5.0	5.0
Neutral	N5. Stirring cereal	3.0	1.0	1.0	5.0	2.0	4.0
Misophonic	M5. Chewing food	4.0	1.0	1.0	1.0	4.0	4.0
Neutral	N6. Woodpecker tapping	4.0	1.0	1.0	1.0	1.0	4.0
Misophonic	M6. Clicking a pen	2.0	4.0	1.0	1.0	1.0	4.0
Pleasant	P7. Wind blowing	1.0	1.0	5.0	1.0	1.0	2.0
Pleasant	P7. Stream flowing	1.0	1.0	1.0	5.0	1.0	2.0

Table S3: Median material and agent property ratings taken across all participants in Experiment 2 (regular sounds) are indicated in each table entry, with rows corresponding to one of the fourteen sounds and columns corresponding to the five materials and one agent. The intended valence category (Neutral, Unpleasant, Misophonic, or Pleasant) is indicted in the far-left column. Materials or agent judged to have the highest likelihood of causing a sound are colored in blue.
