# Supplementary material for: Identification of Everyday Sounds Affects Their Pleasantness
Source: Front Psychol. 2022 Jul 8;13:894034. doi: 10.3389/fpsyg.2022.894034 (PMC9347306; doi:10.3389/fpsyg.2022.894034)
Supplement: Supplementary file 1 [file Data_Sheet_1.ZIP › Supplemental Material/TableS5.pdf]

Sound Name	Identification Accuracy	Rating when perceived as Unpleasant or Misophonic	Identification Accuracy	Rating when perceived as Neutral or Pleasant	Rating Difference
Tool Scraping	Incorrect	-2	Correct	-1	1
Cereal Stirring	Incorrect	-1	Correct	1	2
Chewing food	Correct	-1	Incorrect	0	1
Fork Scraping Plate	Correct	-3	Incorrect	1.5	4.5
Average		-1.75		0.375	

Table S5: Pleasantness ratings for sounds in Experiment 1 as a function of how they are identified. For each sound stimulus, the sound token name is presented in the first column. The Identification Accuracy column illustrates when participants identified the sound in the right emotional category (correct) or when they identified the sound in the wrong emotional category (incorrect). The median pleasantness rating is given for each sound for when it is correctly or incorrectly identified in the correct emotional category. The green and purple color code for these averages connects with the one seen in Table S4 and S6.
