# Supplementary material for: Identification of Everyday Sounds Affects Their Pleasantness
Source: Front Psychol. 2022 Jul 8;13:894034. doi: 10.3389/fpsyg.2022.894034 (PMC9347306; doi:10.3389/fpsyg.2022.894034)
Supplement: Supplementary file 1 [file Data_Sheet_1.ZIP › Supplemental Material/TablesS4.pdf]

ACTUAL SOUND \ PERCIEVED AS	N1. Tool scraping	U1. Fork scraping plate	N2. Ringing church bells	U2. Ringing fire alarm	N3. Squeezing spray bottle	M3. Nose sniffling	N4. Sink draining	M4. Slurping beverage	N5. Stirring cereal	M5. Chewing food	N6. Woodpecker tapping	M6. Clicking a pen	P7. Wind blowing	P7. Stream flowing
N1. Tool scraping	0.62	0.38												
U1.Fork scraping plate	0.08	0.90							0.03					
N2. Ringing church bells			1.00											
U2. Ringing fire alarm			0.03	0.97										
N3. Squeezing spray bottle	0.05				0.95									
M3. Nose sniffling						1.00								
N4. Sink draining							0.90	0.05	0.03					0.03
M4. Slurping beverage								1.00						
N5. Stirring cereal	0.05						0.08	0.05	0.64	0.13		0.03		0.03
M5. Chewing food	0.23	0.03			0.03				0.05	0.67				
N6. Woodpecker tapping											1.00			
M6. Clicking a pen												1.00		
P7. Wind blowing													1.00	
P7. Stream flowing							0.03							0.97

Table S4: Confusion matrix with identification percentages for each non-vocoded sound token identified in Experiment 1. Green boxes denote when a sound in Negative valence group (Unpleasant or Misophonic category) were misidentified as a Neutral sound. Purple boxes denote when a sound in a Neutral valence group were misidentified as a Negative valence group (Unpleasant or Misophonic emotional category). Each sound token has a pair label in their sound name as well.
